# Identifying niche and fitness dissimilarities in invaded marine macroalgal canopies within the context of contemporary coexistence theory

**DOI:** 10.1038/s41598-019-45388-5

**Published:** 2019-06-19

**Authors:** Graham Epstein, Stephen J. Hawkins, Dan A. Smale

**Affiliations:** 1 Marine Biological Association of the United Kingdom, The Laboratory, Citadel Hill, Plymouth PL1 2PB UK; 2 Ocean and Earth Science, University of Southampton, National Oceanography Centre Southampton, Waterfront Campus, European Way, Southampton, SO14 3ZH UK

**Keywords:** Community ecology, Invasive species

## Abstract

Contemporary coexistence theory provides a framework for predicting invasiveness and impact of Invasive Non-Native Species (INNS) by incorporating differences in niche and fitness between INNS and co-occurring native species. The widespread invasive kelp *Undaria pinnatifida* is considered a high-risk INNS, although a robust evidence base regarding its invasiveness and impact is lacking in many regions. Invaded macroalgal canopies at nine coastal sites in the southwest UK were studied over three years to discern whether *Undaria* is coexisting or competing with native canopy-forming species across different habitat types. Spatial, temporal and depth-related trends in species distributions and abundance were recorded within intertidal and subtidal rocky reef as well as on marina pontoons. A primary succession experiment also examined competitive interactions between species. In rocky reef habitats, *Undaria* had lower fitness compared to long-lived native perennials, but was able to coexist due to niche dissimilarity between species. In contrast, *Undaria* was likely to be competing with short-lived native annuals on rocky reef due to large niche overlap and similar fitness. In marina habitats, *Undaria* dominated over all other canopy formers due to low niche diversification and higher fitness. Generalisations on INNS impact cannot be made across habitats or species, without considering many abiotic factors and biotic interactions.

## Introduction

The earliest works on invasion ecology predicted that the most successful invasive non-native species (INNS) would be taxonomically or functionally distinct from the recipient communities to which they are introduced^1,2^. This links to classic niche theory^2–4^, whereby a newly arriving species is predicted to be more successful if it occupies a vacant or under-used niche^5^. More recent theories in invasion ecology, based on factors such as resource utilisation and species diversity, are fundamentally linked to this ‘niche-space’ concept^5–8^. Niche theory does not, however, take into account that in order for an INNS to become successfully established within a recipient community, some overlap of potential niches will inevitably be present in terms of habitat, climate and other abiotic factors where native and non-native species co-occur^9^.

The invasiveness of a species (i.e. its potential to rapidly colonize large and/or multiple areas) is not necessarily linked to its ecological impact^10^. This is supported by niche theory, as competition with native species (and therefore potential for impact) is more likely to occur between functionally similar species or those occupying a similar niche, whereas niche dissimilarity would promote invasiveness but not necessarily competition^11,12^. Contemporary coexistence theory provides a framework for explaining both the invasiveness and impact of INNS at a given site by considering both niche and fitness differences between the invader and co-occurring native species^13–15^. Differences in potential niche promote coexistence, and can be quantified by the degree to which population growth is limited by intraspecific, rather than interspecific, competition. Fitness differences drive competition and are based on disparate traits between species which allow the population of one species to expand to the detriment of, or at a faster rate than, a co-occurring species, potentially leading to displacement^14^. It is the balance between these two continuous variables which determines to what extent an INNS coexists with native species, is excluded by native communities, or becomes dominant^13^.

A coexistence invasion framework can be most easily conceptualised spatially across a stable environment. Following an introduction event, an INNS will spread into a given number of microhabitats and proliferate at varying densities based on its potential niche, the niche diversity across a site and fitness differences between co-occurring species; thus eventually leading to a stable coexisting or dominant climax community^14^. Often called ‘the storage effect’, coexistence can also be mediated by temporal variation, whereby changes in the abiotic or biotic environment lead to fluctuating fitness differences between species and therefore coexistence^16^. INNS are often considered ‘passengers of environmental change’, requiring disturbance or degraded ecosystems to establish and spread^17^. This concept is derivative of the storage effect whereby certain INNS require fluctuations in the abiotic environment in order to coexist or proliferate within native communities^18^.

The application of coexistence theory has largely been constrained to terrestrial plant invasions^15^, rarely being considered within marine ecosystems^19,20^. The widespread invasive kelp *Undaria pinnatifida* (hereafter referred to as *Undaria*), native to the northwest Pacific, is now found in parts of the northeast and southwest Atlantic, southwest and east Pacific, and the Tasman Sea^21^. In many parts of its non-native range, *Undaria* coexists with native canopy-forming macroalgae on natural substrates (i.e. shallow rocky reefs). However, it is generally recorded at highest abundance where the cover or density of native canopy-forming macroalgae are reduced or absent, often due to abiotic factors such as depth or tidal height^22,23^, wave exposure^24^, reduced salinity^25^ or substrate type, aspect, and stability^23,24,26–28^.

Such niche dissimilarity has been supported by some manipulative experiments where the removal of native canopies did not lead to *Undaria* recruitment^29^ or, conversely, when the addition or removal of *Undaria* had no effect on its native counterparts^30–33^. In the majority of cases, however, removal or disturbance of native canopies promotes the recruitment and growth of *Undaria*^31,34–37^. Such promotion suggests that *Undaria* occupies an overlapping niche with native canopy-formers, but has lower fitness. This theory is further supported by post-disturbance recovery patterns, where declines in *Undaria* and increases in native species tend to occur over time^35–37^.

There are some site specific-cases, however, where these generalisations do not apply. Where native canopy diversity is naturally low, *Undaria* may have impacts upon native macroalgal communities or inhibit their recovery on natural rocky substrates e.g. low diversity sites in Argentina^38,39^. Furthermore, although not conclusive, *Undaria* may have higher fitness than native canopy-formers on artificial substrates (i.e. man-made structures such as marina pontoons, pilings and port walls), where it can proliferate with or without disturbance to native macroalgae and become the dominant canopy-former^24,26,28,40–43^. A better understanding of the drivers of *Undaria* invasions is needed to predict the potential for ecological impact on native communities and the need for targeted management^44^.

**Figure 1:**
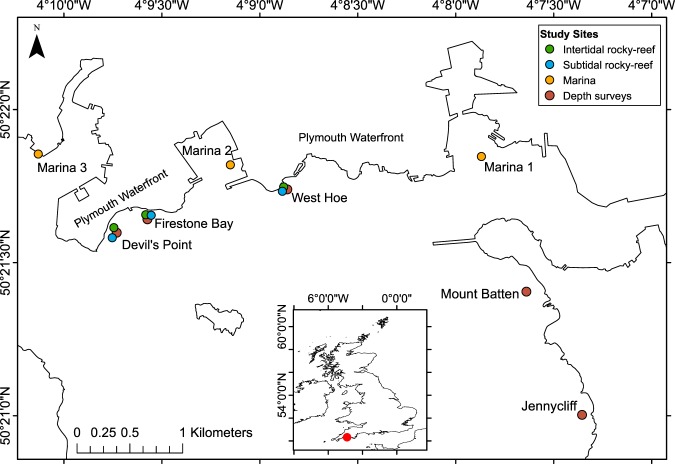
Study sites in Plymouth Sound (location of Plymouth within the UK shown as red point on inset map). Nine monitoring study sites, three each of intertidal rocky reef (green points) subtidal rocky reef (blue points) and marinas (orange points), were selected in March-April 2016. Depth surveys (brown points) were completed in July 2017 at reef monitoring sites and two additional sites (Mount Batten and Jennycliff).

We monitored and manipulated invaded macroalgal canopies over three years to examine the extent to which *Undaria* coexists or competes with native canopy-forming species. Spatial (across both sites and depths) and temporal patterns in species distributions, density and biomass were recorded at multiple sites in Plymouth Sound (UK) representing three distinct habitat types: intertidal rocky reef, shallow subtidal rocky reefs and marina pontoons (Fig. 1). Furthermore, primary succession patterns were examined at both a marina and intertidal reef site to better elucidate competitive interactions between species. Our hypothesis was that spatial variation both across and between complex marine habitats will drive niche and fitness differences and determine distribution-abundance patterns of the invasive kelp and co-occurring native canopy forming macroalgae.

## Results

### Multi-year monitoring

**Figure 2:**
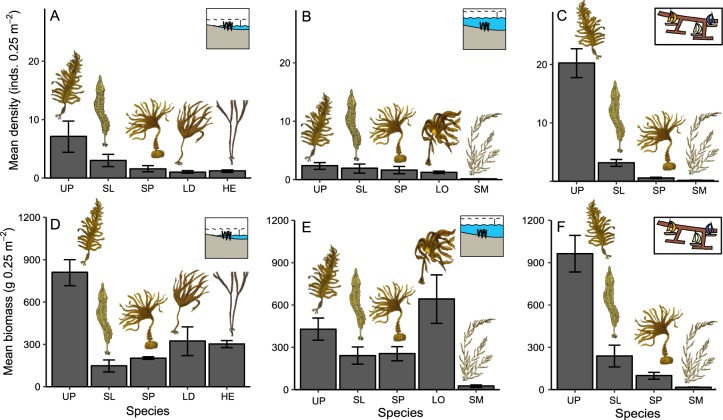
Mean density (**A**–**C**) and biomass (**D**–**F**) (±standard error between years) of canopy forming macroalgae averaging across the three sites and sampling events in June 2016, 2017 and 2018 for each habitat separately (**A**,**D** = intertidal reef; B, E = subtidal reef; C, F = marina pontoons). UP = *U*. *pinnatifida*, SL = *S*. *latissima*, SP = *S*. *polyschides*, LD = *L*. *digitata*, HE = *H*. *elongata*, LO = *L*. *ochroleuca*, SM = *S*. *muticum*. Macroalgae drawing courtesy of Graham Epstein and Jack Sewell.

**Figure 3:**
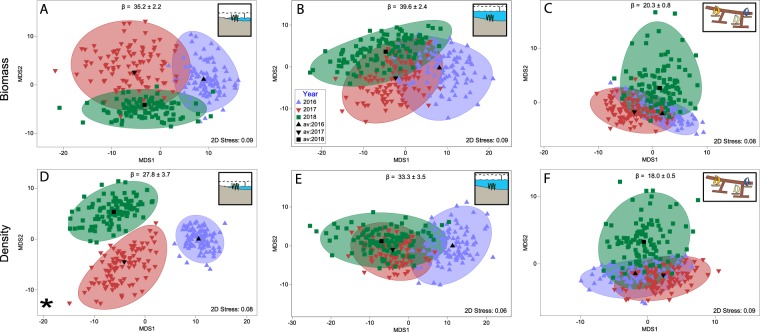
Threshold metric multi-dimensional scaling (tmMDS) plots of bootstrapped average monitoring data within each year of study (blue triangle = 2016, red triangle = 2017, green square = 2018). Biomass (**A**–**C**; g 0.25 m^−2^) and density (**D**–**F**; inds. 0.25 m^−2^) data for each habitat assessed separately (**A**,D = intertidal reef; B, E = subtidal reef; C, F = marina pontoons). Circular areas indicate the 95% confidence region around the bootstrap average. Asterisks indicate significant difference between years based on PERMANOVA (Table S1). β = proxy of mean beta-diversity for each habitat (±SE) measured by multivariate dispersion.

Considering all three sampling years together, intertidal macroalgal canopies were dominated by *Undaria* in June, with it contributing on average the highest density and biomass of any species (Fig. 2). There was also a relatively high biomass of *H*. *elongata* and *L*. *digitata*, and a high density of *S*. *latissima* (Fig. 2). The subtidal rocky reef had the most mixed canopy in June, with *L*. *ochroleuca* being dominant by biomass but with *Undaria*, *S*. *latissima* and *S*. *polyschides* being present in similar densities and only moderately lower in biomass (Fig. 2). There was also a small amount of the non-native *S*. *muticum* found at two of the three subtidal sites (Figs 2, S1). Marinas were dominated by *Undaria* in terms of both biomass and density (Fig. 2), which was interspersed with the native canopy-forming macroalgae *S*. *latissima* and *S*. *polyschides*, and small amounts of the non-native *S*. *muticum* (Fig. 2). There was very low variation in the density and biomass of species between years across the three June sampling events (Fig. 2), although within-year seasonal variability was pronounced (Fig. S1). Across all habitats *Undaria* and *S*. *polyschides* had a clear annual cycle with peak abundance predominantly occurring in June and September, respectively; albeit with some variation between sites. The perennial species *S*. *latissima* exhibited a similar annual pattern but with higher variation both between sites and habitats (Fig. S1), whereas *Laminaria* species on rocky reefs had low variability both seasonally and between sites (Fig. S1). Mean β-diversity (between quadrats) was highest in subtidal habitats and lowest in marinas (Fig. 3). The overall composition of macroalgal canopies did not vary markedly across the three years surveyed within any habitat type (Fig. 3). However, significant between-year variability in assemblage structure (based on count data only) was recorded within intertidal reef habitat, with 2016 being dissimilar to other survey years in 2017 and 2018 (Fig. 3, Tables S1, S2). This was due to lower contributions of *Undaria* and *S*. *latissima* to total density, and higher contributions of *L*. *digitata*, *H*. *elongata* and *S*. *polyschides* (Table S3).

There were significant spatial trends between *Undaria* and co-occurring species in rocky reef habitats. *Undaria* density and biomass had a significant negative correlation with the *Laminaria* species in both intertidal and subtidal habitats (Table 1). *Undaria* biomass was also negatively related to *S*. *latissima* in both habitats, although this was only statistically significant for subtidal biomass (Table 1). For all other species in reef habitats the relationships were less well defined. In the subtidal reef habitat there was a positive relationship between the density of *Undaria* and *S*. *muticum*, on intertidal reef *H*. *elongata* was negatively related to both *Undaria* density and biomass, and *S*. *polyschides* was positively related to *Undaria* density and biomass in both habitats; however, only *Undaria* and *S*. *muticum* on subtidal reef was statistically significant (Table 1). In marina habitats the density and biomass of *Undaria* was not significantly related to any other species, although the positive relationship in biomass between *Undaria* and *S*. *polyschides* was near-significant (Table 1).

**Table 1:** GLMMS and LMMs identifying the influence of co-occurring canopy-forming macroalgae on the density and biomass of *Undaria* within each habitat. The coefficient estimates (Est) and p-value (*p)* are shown for each species along with t-values (t) from LMMs for biomass [log(g + 1 m^−2^)] and z-values (z) from GLMMs for density data (inds. 0.25 m^−2^). Significant coefficients shown in bold (ɑ = 0.01 for biomass, ɑ = 0.05 for density).

Species	Biomass			Density		
	Est	t	*p*	Est	z	*p*
***Intertidal reef***						
*S*. *polyschides*	4.9 × 10^−4^	0.87	0.386	0.009	5.95	0.819
*S*. *latissima*	−1.9 × 10^−3^	−2.28	0.025	0.035	0.23	0.090
*L*. *digitata*	**−2**.**2** × **10**^**−3**^	**−8**.**11**	<**0**.**001**	**−0**.**186**	**1**.**70**	<**0**.**001**
*H*. *elongata*	−6.8 × 10^−4^	−1.53	0.130	−0.114	−1.95	0.051
***Subtidal reef***						
*S*. *polyschides*	2.3 × 10^−4^	0.60	0.550	0.058	1.08	0.281
*S*. *latissima*	**−1**.**8** × **10**^**−3**^	**−3**.**87**	<**0**.**001**	−0.031	−0.82	0.410
*L*. *ochroleuca*	**−2**.**2** × **10**^**−3**^	**−10**.**28**	<**0**.**001**	**−0**.**302**	**−2**.**50**	**0**.**013**
*S*. *muticum*	1.1 × 10^−3^	−0.64	0.526	**0**.**468**	**2**.**15**	**0**.**032**
***Marina***						
*S*. *polyschides*	5.2 × 10^−4^	2.44	0.017	0.020	0.49	0.623
*S*. *latissima*	−1.3 × 10^−4^	−0.81	0.421	<0.001	<0.01	0.998
*S*. *muticum*	−1.3 × 10^−3^	−1.46	0.147	−0.145	−1.57	0.117

### Depth-related trends

**Figure 4:**
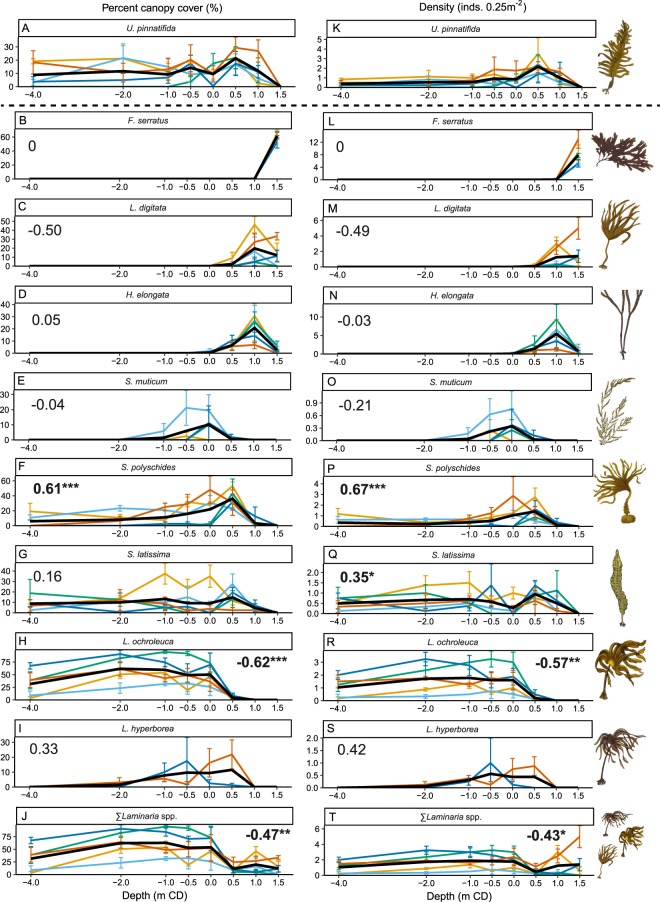
Distribution of canopy-forming macroalgae across depth, in reef habitats of Plymouth Sound. Mean percent canopy cover (**A**–**J**) and density (**K**–**T**) of each species shown ± standard error at each of five sites (orange = Devil’s Point, light blue = Firestone Bay, green = Jennycliff, dark blue = Mount Batten, red = West Hoe). Black lines indicate average across sites. Correlation coefficients with *Undaria* shown for every co-occurring species as well as for the sum of all *Laminaria* spp. Significant correlations shown in bold; asterisks indicate significance level (*=<0.05, **=<0.01, ***<0.001). Macroalgae drawing courtesy of Graham Epstein and Jack Sewell.

On rocky reef *Undaria* was found at depths ranging from + 1 to −4 m CD, with highest density and percent cover in the low intertidal to shallow subtidal zone, and a peak at + 0.5 m CD (Fig. 4). Above + 1 m CD canopy-forming macroalgal assemblages were composed of *F*. *serratus* and *L*. *digitata* only, with *Undaria* completely absent (Fig. 4). Within its depth range *Undaria* co-occurred with seven species of canopy-forming brown macroalgae: *L*. *digitata*, *H*. *elongata*, *S*. *muticum*, *S*. *polyschides*, *S*. *latissima*, *L*. *ochroleuca*, *L*. *hyperborea*. The peak in *Undaria* density/cover at + 0.5 m CD coincided with the lowest density and percentage cover of *Laminaria* species at any depth (Fig. 4). A weak positive correlation was recorded between *S*. *latissima* and *Undaria* across depth; however, this was only significant for count data (Fig. 4). *Undaria* had a strong and significant positive correlation with *S*. *polyschides* with similar distribution patterns across depth, but a strong negative correlation with *L*. *ochroleuca* (Fig. 4). There was also a negative correlation between *Undaria* and *L*. *digitata*, although this was not statistically significant (Fig. 4). When considering the sum of all *Laminaria* species together, *Undaria* again exhibited a significant negative correlation across depths (Fig. 4). It should be noted however, that *L*. *hyperborea* had a non-significant but positive correlation with *Undaria*. This reverse relationship is most likely to be due insufficient data as *L*. *hyperborea* was recorded at extremely low densities across the entire study area.

### Primary succession

**Figure 5:**
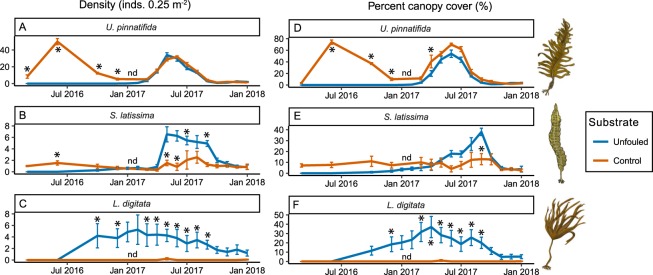
Marina primary succession manipulation - change in density (**A**–**C**) and percent cover (**D**–**F**) of canopy-forming macroalgae following introduction of new substrate into a marina habitat. Means shown ± standard error. Asterisks indicate pairwise differences between old (red) and new unfouled (blue) substrate at each sampling event (Tables S4 and S5). “nd” indicates where data from old substrates was missing so pairwise tests could not be calculated. Macroalgae drawing courtesy of Graham Epstein and Jack Sewell.

Following the introduction of new unfouled substrate into an *Undaria* dominated marina in March 2016, initial colonisation by canopy-forming macroalgae (Oct 16 to Mar 17) was dominated by *L*. *digitata* with low levels of *S*. *latissima* (Fig. 5). The density and cover of *L*. *digitata* were significantly higher compared with control areas on older pontoon substrates, whereas values for *S*. *latissima* were comparable between new and control substrates (Fig. 5). The first *Undaria* recruits were recorded one year following the installation of the new pontoons. The density of *Undaria* increased rapidly from Mar – Jun 17 and did not differ significantly from the control areas for the remainder of the experiment. The cover of *Undaria* also increased rapidly but remained marginally lower on new pontoons compared with control areas, although this variability was statistically significant at only one sampling event (Fig. 5). During this period, the density and cover of *L*. *digitata* on the new pontoons declined, and during the last four months of monitoring were no longer significantly different to values recorded on the older pontoons (Fig. 5). The density and cover of *S*. *latissima* increased on new pontoons during the same period, and was significantly higher than that on older substrates (Fig. 5). However, density and cover subsequently declined and for the last four months of monitoring both metrics were similar between treatments (Fig. 5).

**Figure 6:**
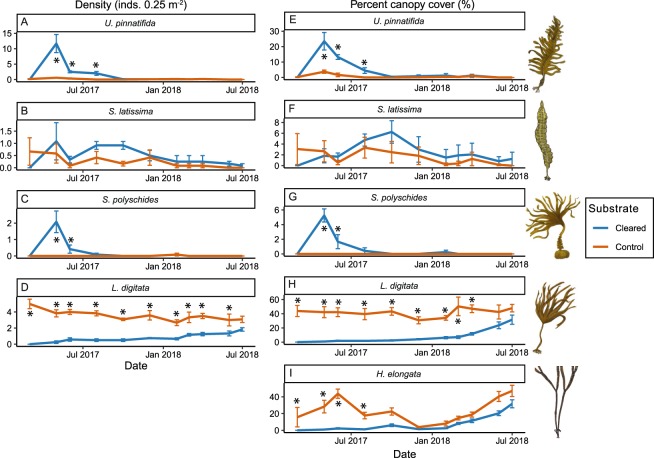
Intertidal reef primary succession manipulation - change in density (**A**–**D**) and percent cover (**E**–**I**) of canopy-forming macroalgae following introduction of cleared substrate into an intertidal reef habitat. Means shown ± standard error. Asterisks indicate pairwise differences between old (red) and new (blue) substrate at each sampling event (Tables S6 and S7). Macroalgae drawing courtesy of Graham Epstein and Jack Sewell.

Cleared substrates within the *L*. *digitata* dominated intertidal rocky reef habitat were quickly colonised by canopy-forming macroalgae after only two months. This early colonisation (May–Aug 17) was dominated by *Undaria*, but also *S*. *polyschides*, both of which were significantly higher in density and cover compared to the control area of undisturbed substrate (Fig. 6). The density and cover of *L*. *digitata* and the cover of *H*. *elongata* on cleared substrates remained low during this period, being significantly lower than values recorded on control plots (Fig. 6). For the remainder of the experiment (Oct 17 to July 18) *Undaria* and *S*. *polyschides* remained at low levels within both cleared and control plots, and no significant differences between treatments were recorded at any sampling point (Fig. 6). During this period there was a sustained increase in both *L*. *digitata* and *H*. *elongata* on new substrates. Even so, density and cover remained significantly lower on cleared compared with undisturbed areas for *L*. *digitata* until the last two months of sampling (Fig. 6). *S*. *latissima* density and cover varied greatly on both cleared and undisturbed areas across the study period, and exhibited no significant differences between substrate types (Fig. 6).

## Discussion

Interpreting results from manipulative, trait-based or demographic studies within the framework of contemporary coexistence theory allows for examination of the extent to which INNS may be coexisting or competing with native species in recipient communities^45^. Given its rapid rate of spread and inconsistencies in its perceived impacts^21^, *Undaria* is a useful model INNS to explore coexistence theory within rarely considered marine ecosystems^15^. *Undaria* has been present within the current study region for at least 15 years, where it now co-occurs with up to ten different species of canopy-forming macroalgae. Overall, there was very low annual variation and no major changes in macroalgal canopy density, biomass or composition over the three years of monitoring. Although inference is somewhat limited due to the duration of this study, the results suggest that large-scale temporal variability and maintenance of coexistence through the storage effect is not a primary driver of assemblage structure. This also indicates that the invader may have reached a relative equilibrium within the recipient community, as none of the species recorded here exhibited significant increases or decreases in abundance over the study period.

We recorded high variation in macroalgal assemblage structure between habitats. In complex rocky reef habitats, *Undaria* was predominantly found in areas that supported lower density and cover of perennial species, and reached relatively low abundances in areas where these species were experimentally removed. Conversely, on uniform marina pontoons, *Undaria* was the dominant species, exhibited no significant spatial relationships with native species and became established as the dominant species following succession on newly introduced substrate. Overall, when considering potential drivers of assemblage structure at the site level, based on these results we suggest that niche diversity primarily promoted coexistence of species on rocky reef, whereas fitness differences governed competition within marinas. It must be noted, however, that at the level of micro-habitats there are complex species-specific patterns which cannot be generalised across a given site. Using coexistence theory to contextualise observational or correlative findings, as shown here, allows for a clearer understanding of INNS distribution patterns and potential community level impacts. These factors should be of primary concern for INNS research, as they will directly contribute to management and conservation priorities.

On natural rocky reef substrates, *Undaria* exhibited a significant negative spatial relationship with the two dominant perennial species in both intertidal (i.e. *Laminaria digitata*) and subtidal habitats (i.e. *Laminaria ochroleuca*). The depth surveys also indicated that *Undaria* predominantly occupies a depth/elevation zone in which *Laminaria* species are found in lower abundance and cover, which may suggest niche differences as the primary driver leading to differing abundance-distribution patterns.

One factor which may drive niche differences and spatial separation between *Undaria* and *Laminaria* spp. is the ability of *Undaria* to grow successfully on a wide range of types of substrate, aspects and stabilities^23,24,27^. This generalist life strategy may allow it to fill a niche that is not occupied by the perennial Laminarians which generally require stable rocky substrates in order to successfully grow and mature^46^. In many cases, however, these species interact on stable rocky substrates, suggesting other drivers influence abundance-distribution patterns.

The primary succession experiment showed that clearance of *L*. *digitata* dominated areas leads to an opportunistic pulse of *Undaria* recruitment, confirming an overlapping niche. This was followed by recovery of *L*. *digitata* and a concurrent decline in *Undaria*, indicating higher fitness in *L*. *digitata*. However, *Undaria* recruited in relatively low densities and cover following the removal of *L*. *digitata*, and it was not able to recruit in the second year after clearances even when there was still significantly lower *Laminaria* density and cover compared to controls. This would suggest that *Undaria* has an overlapping but distinct niche when compared to *L*. *digitata*, and where they do overlap *Undaria* has lower fitness.

Due to differences in tolerance to desiccation and temperature stress, the two *Laminaria* species within this study region form two relatively distinct zones on rocky reefs; *L*. *digitata* on intertidal reef and *L*. *ochroleuca* on subtidal reef^47^. Although there is evidence of relatively high desiccation tolerance in *Undaria* sporophylls and gametophytes, information is lacking on the tolerance of growing blade tissue^48^. Due to its inability to successfully proliferate in cleared areas of the *L*. *digitata* zone, we suggest that *Undaria* has lower resistance to desiccation and temperature stress than *L*. *digitata*. However, *Undaria* is found at highest density and biomass at elevations above the *L*. *ochroleuca* zone, potentially indicating an intermediate tolerance between the two *Laminaria* species and allowing it to occupy a depth niche between the two Laminarians.

This does not, however, explain why *L*. *digitata* is not outcompeting *Undaria* and dominating on stable rocky substrate in the low intertidal to shallow subtidal fringe. In some regions *L*. *digitata* can be found to depths of up to 15 m, dependent on competition, wave exposure, light, temperature and grazing pressure^49,50^. While it is feasible that any of these factors could influence observed abundance-distribution patterns in the current study, *L*. *digitata* can be present in a wide range of wave exposures^50^. ‘Top-down’ grazing pressure is also not thought to be of major importance to kelp population structure along most of the UK coastline^49,51^, except at its deeper extent^52^. *L*. *digitata* is, however, known to require relatively high light levels to reproduce and grow^53,54^, whereas *Undaria* is able to persist under a wide range of light climates^21,55^. In the relatively turbid conditions of Plymouth Sound, it may be that *L*. *digitata* becomes light limited in the deeper intertidal-subtidal fringe (which is generally immersed in 1–5 m of turbid seawater due to the tidal range in the region), allowing *Undaria* to occupy this vacant space. Further manipulations of assemblages within the intertidal fringe would be needed to fully determine the niche versus fitness differences between these species.

It is also necessary to consider why *Undaria* is not proliferating in the lower subtidal areas which are currently *L*. *ochroleuca* dominated. One potential driver could be the comparatively low rate of nutrient uptake and nitrate storage in *Undaria* when compared to Laminarians^21,56^. Increased water motion enhances nutrient uptake in kelps^57^ and can enhance the growth rate of *Undaria*^58^. As tidal water flow is likely to be higher in the subtidal fringe when compared to the lower subtidal zone, *Undaria* may have lower fitness than *L*. *ochroleuca* in lower velocity subtidal waters. Although not possible as part of this study due to logistical challenges in experimental design, providing cleared or clean substrate in lower subtidal areas and assessing the colonisation and succession of these two species would allow identification of potential niche versus fitness differences between *Undaria* and *L*. *ochroleuca*.

Overall, these results support studies from other regions which suggest that *Undaria* has lower competitive ability when compared to native canopy dominant perennials^21,59^, referencestherein. The results also highlight that the persistence of *Undaria* in many settings is likely to be due to its relatively broad niche, allowing it to occupy underused resources or vacant space to coexist with native perennials at the site level.

On rocky reef, both *Undaria* and the native annual canopy-forming species *S*. *polyschides* were strongly correlated across depth and showed similar responses in the primary succession experiments. This would suggest that these species occupy a similar niche throughout rocky reefs in the study region. Both species are annual, relatively opportunistic and are found at highest abundance and cover in the subtidal fringe^22,24,60,61^. They also recruit, reach maximum biomass and senesce at similar times of year^60–62^. Therefore, it has previously been suggested that these two species occupy a similar niche and may directly compete for space or resources^22,24,50,60^. Species which occupy the same or highly similar niche while having little to no fitness dissimilarities would be expected to have strong positive spatial relationships across a given site. However, in this study, neither the density nor biomass of *S*. *polyschides* showed a significant spatial relationship with *Undaria* in either intertidal or subtidal reef habitats. This would suggest that there are more complex competitive interactions occurring which does not allow full coexistence between these species.

If *Undaria* was found to be outcompeting and potentially displacing *S*. *polyschides*, it would be pertinent to consider how this may alter wider ecosystem functioning. Previous studies have shown that these two species harbour a similar epifaunal and epifloral assemblages and therefore substitution of the species may have minimal community-level impacts^63^. Moreover, at the wider regional scale *Undaria* is far less tolerant of wave action than *S*. *polyschides*, which can dominate under wave-exposed conditions^24,64^, suggesting that competitive exclusion could only occur at wave sheltered sites and that regional displacement of *S*. *polyschides* is highly unlikely. However, further research would be needed to determine wider ecological consequences such as trophic provision to grazers, habitat provision to mobile species, primary production and carbon cycling^49^.

In comparison to reef habitats, the environment within marinas is relatively homogenous and as such niche diversity is reduced. The relative homogeneity in community structure was highlighted by the low β-diversity recorded on marina pontoons when compared to reef habitats. The pontoons available for sampling in this study were all of uniform substrate, depth and exposure, and were located adjacent to one another, with similar exposure, light availability, water flow and temperature. It is likely, therefore, that relationships in distribution-abundance patterns between species across a given site will be strongly influenced by fitness differences and competitive interactions. In all three marinas *Undaria* was the dominant species in terms of both density and biomass (Fig. S1); it also exhibited no spatial or temporal correlations with co-occurring species. This may indicate that *Undaria* is able to reach similar population size or density at varying levels of interspecific competition from co-occurring species, however further evidence would be needed to confirm this. The primary succession experiment which showed that newly-available substrate introduced into *Undaria* dominated marinas can lead to a significantly higher recruitment of native canopy-forming species (*L*. *digitata* and *S*. *latissima*), both of which were eventually replaced by *Undaria*, also suggests fitness differences as the primary driver.

Marinas are generally located in areas of intense human activity with modified abiotic conditions. Marina environments are often characterised by low salinity and high turbidity, pollution and nutrient levels, which generally support distinct and often depauperate epifaunal and epifloral communities that lack long-lived native species compared to adjacent natural habitats^65^. The higher fitness of *Undaria* when compared to native canopy-formers in marinas may in part be due to its ability to tolerate more extremes in environmental variables^4,40,41,62^. Perhaps the largest abiotic dissimilarity between marina pontoons and rocky reef habitats is the constant shallow depth in which floating pontoons are maintained when compared to tidal rocky habitats. One consequence of this is that macroalgae are subjected to constant high light intensities, which often leads to substantial biofouling and eventual detachment or smothering of blade tissue^66,67^. Here, *Undaria* plants were observed to support significantly less epiphytic loading compared with native species, potentially due to its fast growth rate and annual senescence, or perhaps the presence of antifouling compounds^66,67^. This may be a further mechanism underpinning the higher fitness of *Undaria* over native perennials within marinas. This study adds further support to the importance of artificial habitats in the invasion pathway of *Undaria* both across regions and locally into adjacent natural habitats^24,42,68^. The optimal habitat for *Undaria* within this study region appears to be floating pontoons in marinas – a habitat that has no natural analogue^69^.

*Undaria* has been reported to have relatively low ecological impact in many locations to which it has been introduced, particularly where it occurs within dense native canopies^21,59^. Although this study largely supports these conclusions, it highlights that there can be many context-specific species interactions which should also be considered before clear conclusions can be drawn. Within this study region, and perhaps across the northeast Atlantic, *Undaria* may exert low community-level impacts within natural habitats, due to its relatively low fitness when compared to dominant native perennial canopy-formers. *Undaria* could, however, influence co-occurring macroalgae within its invaded niche, potentially displacing functionally similar native species. Further targeted research would be needed to better quantify both lethal and sub-lethal effects of *Undaria* on species within its niche.

The distribution, proliferation and potential impact of INNS is highly dependent on complex niche and fitness differences between individual species, which themselves can vary across habitats. The quantification of INNS impact is therefore wholly dependent on the response metrics recorded and the spatial scale to which conclusions are drawn^20,70^. This is particularly evident within complex marine habitats dominated by, for example, macroalgal canopy-formers, which interact across multiple spatial and temporal scales, occupying distinct yet overlapping microhabitats and niches. For management purposes INNS are often ranked in terms of their broad-scale impact on the natural environment, which may occur at a continental or even global scale^71,72^. More consideration must be given to the context-specific nature of INNS impacts prior to wider scale management decisions.

## Methods

### Site selection

*Undaria* was first recorded in Plymouth Sound (southwest UK) in 2003. Since then it has successfully colonised both artificial and natural substrates in intertidal and shallow subtidal habitats^24,28,34,63^. Here it interacts with a range of native canopy forming macroalgae, including both perennial and annual species, although its impacts on native assemblages are poorly understood^21,50^.

Within Plymouth Sound, study sites were chosen based on the presence of: 1) available safe access points; 2) approval for scientific work; 3) widespread occurrence of *Undaria* (based on previous information or *in situ* sightings); 4) similar substrate within habitat types; 5) extensive suitable substrate. Nine monitoring sites were selected randomly from a larger pool between 10th March and 5th April 2016. Searches of the low intertidal zone were made across the Plymouth waterfront on low spring tides, subtidal searches were made at seven sites across the same area, and site visits were made at four marina sites (Fig. 1). Three marina and reef sites were selected, with subtidal sites deeper and adjacent to intertidal sites (Fig. 1). All marinas were within sheltered, non-drying harbours, with similarly constructed concrete pontoons. The intertidal and subtidal reef sites were all sheltered to moderately-sheltered from wave action, being characterised by extensive bedrock platforms interspersed with areas of larger boulders and compacted cobbles. Two additional reef sites were selected at the eastern end of the Plymouth waterfront to increase replication for depth profile surveys (Fig. 1). Manipulative primary succession experiments were established at one marina (Marina 1) and one intertidal site (Devil’s Point), adjacent to two of the monitoring sites.

### Multi-year monitoring

Macroalgal canopies were surveyed in June 2016, 2017 and 2018. All nine monitoring sites (Marinas: 1–3; Intertidal rocky-reef: Devil’s Point, Firestone Bay, West Hoe; Subtidal rocky-reef: Devil’s Point, Firestone Bay, West Hoe) were sampled over a two week period at each sampling event. Although *Undaria* can be found throughout the year in the UK^69^ it has an annual life-history, predominantly being found in late spring to early summer^26,60,63,69^ when sampling intensity was concentrated. Sampling in September and December 2016 and March 2017 explored seasonal patterns in density and biomass. This was not used in formal analyses and is used for qualitative comparisons only.

As *Undaria* is predominantly found in low intertidal to shallow subtidal habitats^24,28,63^, subtidal sites were restricted to depths of 0.5–1.2 m below chart datum (CD) and intertidal sites to 0.3–1 m above CD. On each survey, ten replicate 0.5 × 0.5 m quadrats (stratified to rocky substrate) were placed haphazardly (each separated by at least 2 m) within an area of approximately 100 m^2^ around a permanent marker at each site. All subtidal sites were sampled using SCUBA, intertidal sites were sampled either with SCUBA or on low-spring tides.

Surveys within marinas were conducted on the immersed vertical sides of floating pontoons at a depth of 0–0.5 m below the surface. Ten replicate 0.5 × 0.5 m quadrats were positioned haphazardly against the pontoon surface. Based on substrate suitability, interactions with vessels and human disturbance, sampling was restricted to approximately 40 m^2^ of pontoon in the outer section of the marina. Due to the relatively limited area available for sampling, the position of each quadrat was noted to avoid overlapping quadrat samples during the study.

All canopy-forming macroalgae (*U*. *pinnatifida*, *Saccharina latissima*, *Laminaria ochroleuca*, *Laminaria digitata*, *Laminaria hyperborea*, *Fucus serratus*, *Fucus vesiculosus*, *Ascophyllum nodosum*, *Saccorhiza polyschides*, *Himanthalia elongata*, *Sargassum muticum*) were destructively sampled from each quadrat by gently prising the holdfast from the substrate, and placed into collection bags for further analysis. The density (inds. 0.25 m^−2^) and biomass (g 0.25 m^−2^) of each species was recorded for each quadrat separately. To concentrate further analyses on those species that have the potential to influence distribution-abundance patterns of *Undaria*, species which contributed less than 1% of the total biomass of the native macroalgal canopies in each habitat type were excluded.

### Depth profiles

In July 2017 depth-related patterns in the density and cover of canopy-forming macroalgae were examined at five reef sites (Fig. 1). At each site four replicate transects (each separated by approximately 8 m) were surveyed from + 1.5 m to −4 m CD by SCUBA. Based on previous experience and preliminary surveys in Plymouth Sound, depths below −4 m CD are dominated by gravel-sandy substrates lacking canopy-forming macroalgae. Therefore greater depths were not included within this survey; however, in some areas canopy-forming macroalgae may be present at such depths, albeit at low density and cover. For each transect the same two surveyors swam 2–3 m apart along a fixed compass bearing, perpendicular to the depth contour. Each surveyor haphazardly placed a 0.5 × 0.5 m quadrat (stratified to rocky substrate) at eight depths across the survey range ( + 1.5, + 1.0, + 0.5, 0, −0.5, −1, −2. −4 CD). These were located using a digital depth meter corrected by estimated tidal height from the POLTIPS-3 software at the median time of the survey. Sampling was non-destructive; within each quadrat both density and cover of canopy-forming macroalgae (species as above) was visually quantified as counts of individual plants and percent canopy-cover per 0.25 m^2^. If no suitable rocky substrate could be found at a given depth along a transect, data (counts and cover) was recorded as missing rather than zero and replication at that depth was reduced. Prior to analysis the paired quadrats were averaged.

### Primary succession

In March 2016 five new unfouled sections of marina pontoon were replaced at Marina 1 (Fig. 1). This novel substrate provided an opportunity to monitor colonisation and primary succession of canopy-forming macroalgae over time. From monthly observations, new recruits were noted in August 2016 and could be identified to species by October 2016. The canopy-forming macroalgae were non-destructively sampled monthly from October 2016 – January 2018 (except for November 2016). At each sampling event, three 0.5 × 0.5 m quadrats were placed haphazardly against the outer side of each of the five pontoon sections. Within each quadrat, the density and cover of each canopy-forming macroalgae species was estimated visually by a single observer. New pontoons were compared to five adjacent pontoon sections which had not been replaced, therefore containing established macroalgal assemblages. These older pontoons (which had been *in situ* for > 10 years) were sampled using the same method from March 2016 – January 2018, with sampling intensity initially at three month intervals, but monthly from March 2017.

A similar manipulation was also conducted at Devil’s Point to compare primary succession between natural and artificial habitats (Fig. 1). Eight circular treatment plots, 2 m in diameter (each separated by at least 2 m), were established within *Laminaria digitata* dominated reef habitat at a tidal height of + 0.8 to + 1.2 m CD. Each plot was randomly assigned as either ‘control’ or ‘clearance’ treatments. A permanent marker was placed in the centre of each plot using a stainless steel screw and coloured labels. In March 2017 all biota were removed from clearance plots in three stages: 1) manual removal by hand, 2) heat treatment of substrate using a Sheen x300 weed control flame gun, 3) secondary heat treatment using the same method 12 days later. Following the clearances, canopy-forming macroalgae were non-destructively sampled (monthly where possible but at least bimonthly) from March 2017 to July 2018. At each sampling event, three 0.5 × 0.5 m quadrats were haphazardly placed in each plot. Counts were made of each canopy-forming macroalgae (except for *H*. *elongata* ‘buttons’ due to time constraints) and the percentage canopy cover of each species was estimated visually by a single observer. For both the marina and intertidal reef, data were averaged within each pontoon/plot at each sampling event before further analysis.

### Data analysis

Using the monitoring data, interannual variability in macroalgal canopy composition was examined for each habitat-type separately using multivariate techniques. Raw data were first converted to proportional values and square root transformed (to down weight the importance of dominant species) prior to analysis. Resemblance matrices were constructed based on Bray-Curtis similarity and visualised using threshold metric multidimensional scaling (tmMDS) on bootstrap averages with their 95% confidence regions. Statistical differences in multivariate canopy structure between years was assessed using PERMANOVA with ‘year’ (three levels, fixed factor), and ‘site’ (three levels, random factor) as factors. Where differences between years were identified, post-hoc tests using pair-wise PERMANOVA, and similarity percentage breakdowns (SIMPER) were used to determine the principal contributors to the observed dissimilarity within significant pairwise contrasts. As a proxy for beta-diversity (β), the PERMDISP routine was used to calculate mean multivariate dispersion between quadrats for each habitat separately. All PERMANOVAs were run with 9999 permutations of residuals under a reduced model with Type 3 (partial) sums of squares. tmMDS plots were visualised using 50 restarts and a minimum stress of 0.01. Bootstrap averages were calculated with 100 bootstraps per group, with automatic selection of dimensions based on ρ > 0.99.

Spatial trends between *Undaria* and co-occurring macroalgae were assessed using linear mixed models (LMM) for biomass data (log transformed due to strong right-skewness and heterogeneity of variances) and negative binomial generalized linear mixed models (nbGLMM) for count data (due to overdispersion from the Poisson distribution). Data from the multi-year monitoring survey were analysed for each habitat separately. In all cases *Undaria* was modelled as a function of the biomass/count of each co-occurring species (treated as additive fixed continuous factors). Both ‘site’ (categorical; three levels) and ‘year’ (categorical; three levels) were also included as random factors in order to discern overall spatial trends across sites and years. Validation of all models was graphical, using diagnostic quantile-quantile plots and predicted versus residual plots. Where transformations or random error structures failed to fully reduce residual structuring or heterogeneity of variances, test statistics were interpreted at a conservative level of ɑ = 0.01 to decrease the probability of Type 1 errors. LMMs and nbGLMMs were fitted using the *lmer* and *glmer*.*nb* commands respectively, both from the *lme4* package in R^73^.

To assess depth-related distribution trends between *Undaria* and co-occurring species, Spearman rank correlation tests were calculated between *Undaria* and each co-occurring species from the depth distribution survey. Data were first averaged across transects within each survey site and only those depths in the range of *Undaria* occurrence were used to calculate correlations. All Spearman correlations and significance tests were calculated with Holm adjusted p-values to account for multiple testing using the *corr*.*test* function from the *psych* package in R^74^.

For the primary succession manipulation, variability in the biomass/count of each species between the cleared (or new) substrate and control areas were examined with two-way analysis of variance (ANOVA). Prior to statistical analysis all count data were log transformed (log[x + 1]) and percent cover data were arcsin transformed (asin√x/100). Values of density (inds. 0.25 m^−2^), and cover for each species were modelled as a function of “date” (categorical; 15 levels for marina, 10 levels for reef) and “substrate” (categorical; two levels: cleared/new, control), with their interaction. The approach to model validation was the same as described above. Significant pairwise differences between substrates at each sampling point were tested using post hoc F-tests with Holm adjusted p-values. ANOVAs were constructed using the *lm* function from base R^75^ and pairwise tests were implemented using the *testInteractions* function from the *phia* package in R^76^. All univariate statistics were implemented in R 3.4.3^75^, multivariate statistics in PRIMER-e version 7^77^, data manipulation used the *dplyr* package^78^, graphs were created using *ggplot2*^79^ and maps (Fig. 1) were made within ArcMap 10.3.1.

## Supplementary information

Supplementary Material

## Data Availability

The datasets generated during and/or analysed during the current study are available from the corresponding author on reasonable request.

## References

[1] Darwin, C. *On the Origin of the Species by Means of Natural Selection: or*, *the Preservation of Favoured Races in the Struggle for Life* (J. Murray, 1859).

[2] Elton, C. S. *The ecology of invasions by animals and plants*. (Springer, 1958).

[3] Hutchinson GE (1959). Homage to Santa Rosalia or Why Are There So Many Kinds of Animals?. The American Naturalist.

[4] Herbold B, Moyle PB (1986). Introduced Species and Vacant Niches. The American Naturalist.

[5] Levine JM, D’Antonio CM (1999). Elton revisited: A review of evidence linking diversity and invasibility. Oikos.

[6] Shea K, Chesson P (2002). Community ecology theory as a framework for biological invasions. *Trends in Ecology &*. Evolution.

[7] Dunstan PK, Johnson CR (2007). Mechanisms of invasions: Can the recipient community influence invasion rates?. Botanica Marina.

[8] Stachowicz JJ, Fried H, Osman RW, Whitlatch RB (2002). Biodiversity, invasion resistance, and marine ecosystem function: Reconciling pattern and process. Ecology.

[9] Lodge DM (1993). Biological invasions: Lessons for ecology. Trends in Ecology & Evolution.

[10] Ricciardi A, Cohen J (2007). The invasiveness of an introduced species does not predict its impact. Biological Invasions.

[11] Ricciardi A, Hoopes MF, Marchetti MP, Lockwood JL (2013). Progress toward understanding the ecological impacts of nonnative species. Ecological Monographs.

[12] Valéry L, Fritz H, Lefeuvre J-C, Simberloff D (2008). In search of a real definition of the biological invasion phenomenon itself. Biological Invasions.

[13] MacDougall AS, Gilbert B, Levine JM (2009). Plant invasions and the niche. Journal of Ecology.

[14] Chesson P (2000). General theory of competitive coexistence in spatially-varying environments. Theoretical population biology.

[15] Barabas G, Andrea RD, Stump SM (2018). Chesson’s coexistence theory. Ecological Monographs.

[16] Chesson PL, Warner RR (1981). Environmental Variability Promotes Coexistence in Lottery Competitive Systems. The American Naturalist.

[17] MacDougall AS, Turkington R (2005). Are invasive species drivers or passengers of change in degraded ecosystems. Ecology.

[18] Britton-Simmons KH, Abbott KC (2008). Short- and long-term effects of disturbance and propagule pressure on a biological invasion. Journal of Ecology.

[19] Hart SP, Marshall DJ (2013). Environmental stress, facilitation, competition, and coexistence. Ecology.

[20] Hannam MP, Wyllie-Echeverria S (2014). Microtopography promotes coexistence of an invasive seagrass and its native congener. Biological Invasions.

[21] Epstein G, Smale DA (2017). *Undaria pinnatifida*: A case study to highlight challenges in marine invasion ecology and management. Ecology and evolution.

[22] Castric-Fey A, Girard A, Lhardyhalos MT (1993). The Distribution of *Undaria pinnatifida* (Phaeophyceae, Laminariales) on the Coast of St. Malo (Brittany, France). Botanica Marina.

[23] Russell LK, Hepburn CD, Hurd CL, Stuart MD (2008). The expanding range of *Undaria pinnatifida* in southern New Zealand: distribution, dispersal mechanisms and the invasion of wave-exposed environments. Biological Invasions.

[24] Epstein G, Smale DA (2017). Environmental and ecological factors influencing the spillover of the non-native kelp, *Undaria pinnatifida*, from marinas into natural rocky reef communities. Biological Invasions.

[25] Bollen M, Pilditch CA, Battershill CN, Bischof K (2016). Salinity and temperature tolerance of the invasive alga *Undaria pinnatifida* and native New Zealand kelps: Implications for competition. Marine Biology.

[26] Cremades J, Freire O, Peteiro C (2006). Biología, distribución e integración del alga alóctona *Undaria pinnatifida* (Laminariales, Phaeophyta) en las comunidades bentónicas de las costas de Galicia (NW de la Península Ibérica). Anales del Jardín Botánico de Madrid.

[27] Sliwa C, Johnson CR, Hewitt CL (2006). Mesoscale dispersal of the introduced kelp *Undaria pinnatifida* attached to unstable substrata. Botanica Marina.

[28] Heiser S, Hall-Spencer JM, Hiscock K (2014). Assessing the extent of establishment of *Undaria pinnatifida* in Plymouth Sound Special Area of Conservation, UK. Marine Biodiversity Records.

[29] Morelissen B, Dudley BD, Phillips NE (2016). Recruitment of the invasive kelp *Undaria pinnatifida* does not always benefit from disturbance to native algal communities in low-intertidal habitats. Marine Biology.

[30] South PM (2015). Transient effects of an invasive kelp on the community structure and primary productivity of an intertidal assemblage. Marine and Freshwater Research.

[31] South PM, Thomsen MS (2016). The ecological role of invading *Undaria pinnatifida*: an experimental test of the driver–passenger models. Marine Biology.

[32] Valentine JP, Johnson CR (2005). Persistence of the exotic kelp *Undaria pinnatifida* does not depend on sea urchin grazing. Marine Ecology Progress Series.

[33] Forrest BM, Taylor MD (2002). Assessing invasion impact: survey design considerations and implications for management of an invasive marine plant. Biological Invasions.

[34] De Leij, R., Epstein, G., Brown, M. P. & Smale, D. A. The influence of native macroalgal canopies on the distribution and abundance of the non-native kelp *Undaria pinnatifida* in natural reef habitats. *Marine Biology* **164**, 10.1007/s00227-017-3183-0 (2017).

[35] Edgar GJ, Barrett NS, Morton AJ, Samson CR (2004). Effects of algal canopy clearance on plant, fish and macroinvertebrate communities on eastern Tasmanian reefs. Journal of Experimental Marine Biology and Ecology.

[36] Thompson GA, Schiel DR (2012). Resistance and facilitation by native algal communities in the invasion success of *Undaria pinnatifida*. Marine Ecology Progress Series.

[37] Valentine JP, Johnson CR (2003). Establishment of the introduced kelp *Undaria pinnatifida* in Tasmania depends on disturbance to native algal assemblages. Journal of Experimental Marine Biology and Ecology.

[38] Casas G, Scrosati R, Piriz ML (2004). The invasive kelp *Undaria pinnatifida* (Phaeophyceae, Laminariales) reduces native seaweed diversity in Nuevo Gulf (Patagonia, Argentina). Biological Invasions.

[39] Irigoyen AJ, Trobbiani G, Sgarlatta MP, Raffo MP (2011). Effects of the alien algae *Undaria pinnatifida* (Phaeophyceae, Laminariales) on the diversity and abundance of benthic macrofauna in Golfo Nuevo (Patagonia, Argentina): potential implications for local food webs. Biological Invasions.

[40] Curiel D, Guidetti P, Bellemo G, Scattolin M, Marzocchi M (2001). The introduced alga *Undaria pinnatifida* (Laminariales, Alariaceae) in the lagoon of Venice. Hydrobiologia.

[41] Farrell P, Fletcher RL (2006). An investigation of dispersal of the introduced brown alga *Undaria pinnatifida* (Harvey) Suringar and its competition with some species on the man-made structures of Torquay Marina (Devon, UK). Journal of Experimental Marine Biology and Ecology.

[42] James K, Shears NT (2016). Proliferation of the invasive kelp *Undaria pinnatifida* at aquaculture sites promotes spread to coastal reefs. Marine Biology.

[43] Veiga P, Torres AC, Rubal M, Troncoso J, Sousa-Pinto I (2014). The invasive kelp *Undaria pinnatifida* (Laminariales, Ochrophyta) along the north coast of Portugal: Distribution model versus field observations. Marine Pollution Bulletin.

[44] Epstein G, Hawkins SJ, Smale DA (2018). Removal treatments alter the recruitment dynamics of a global marine invader - Implications for management feasibility. Marine Environmental Research.

[45] HilleRisLambers J, Adler PB, Harpole WS, Levine JM, Mayfield MM (2012). Rethinking Community Assembly through the Lens of Coexistence Theory. Annual Review of Ecology, Evolution, and Systematics.

[46] Kain JM (1962). Aspects Of the biology of Laminaria hyperborea I. vertical distribution. Journal of the Marine Biological Association of the United Kingdom.

[47] King Nathan G., Wilcockson David C., Webster Richard, Smale Dan A., Hoelters Laura S., Moore Pippa J. (2017). Cumulative stress restricts niche filling potential of habitat-forming kelps in a future climate. Functional Ecology.

[48] Bollen M, Battershill CN, Pilditch CA, Bischof K (2017). Desiccation tolerance of different life stages of the invasive marine kelp Undaria pinnatifida: Potential for overland transport as invasion vector. Journal of Experimental Marine Biology and Ecology.

[49] Smale DA, Burrows MT, Moore P, O’Connor N, Hawkins SJ (2013). Threats and knowledge gaps for ecosystem services provided by kelp forests: a northeast Atlantic perspective. Ecology and evolution.

[50] Yesson C, Bush LE, Davies AJ, Maggs CA, Brodie J (2015). The distribution and environmental requirements of large brown seaweeds in the British Isles. Journal of the Marine Biological Association of the United Kingdom.

[51] Hereward HFR, Foggo A, Hinckley SL, Greenwood J, Smale DA (2018). Seasonal variability in the population structure of a habitat-forming kelp and a conspicuous gastropod grazer: Do blue-rayed limpets (Patella pellucida) exert top-down pressure on Laminaria digitata populations?. Journal of Experimental Marine Biology and Ecology.

[52] Jones NS, Kain JM (1967). Subtidal algal colonization following the removal of *Echinus*. Helgoländer wissenschaftliche Meeresuntersuchungen.

[53] Han T, Kain JM (1996). Effect of photon irradiance and photoperiod on young sporophytes of four species of the Laminariales. European Journal of Phycology.

[54] Delebecq G (2013). Influence of local environmental conditions on the seasonal acclimation process and the daily integrated production rates of Laminaria digitata (Phaeophyta) in the English Channel. Marine Biology.

[55] Morelissen B, Dudley BD, Geange SW, Phillips NE (2013). Gametophyte reproduction and development of *Undaria pinnatifida* under varied nutrient and irradiance conditions. Journal of Experimental Marine Biology and Ecology.

[56] Dean PR, Hurd CL (2007). Seasonal growth, erosion rates, and nitrogen and photosynthetic ecophysiology of *Undaria pinnatifida* (heterokontophyta) in southern New Zealand. Journal of Phycology.

[57] Gerard VA (1982). *In situ* water motion and nutrient uptake by the giant kelp *Macrocystis pyrifera*. Marine Biology.

[58] Nanba N (2011). Effect of water flow velocity on growth and morphology of cultured *Undaria pinnatifida* sporophytes (Laminariales, Phaeophyceae) in Okirai Bay on the Sanriku coast, Northeast Japan. Journal of Applied Phycology.

[59] South PM, Floerl O, Forrest BM, Thomsen MS (2017). A review of three decades of research on the invasive kelp *Undaria pinnatifida* in Australasia: An assessment of its success, impacts and status as one of the world’s worst invaders. Marine Environmental Research.

[60] Fletcher RL, Farrell P (1999). Introduced brown algae in the North East Atlantic, with particular respect to *Undaria pinnatifida* (Harvey) Suringar. Helgolander Meeresuntersuchungen.

[61] Norton T, Burrows E (1969). Studies on marine algae of the British Isles. 7. *Saccorhiza polyschides* (Lightf.) Batt. British Phycological Journal.

[62] Floc’h JY, Pajot R, Wallentinus I (1991). The Japanese brown alga *Undaria pinnatifida* on the coast of France and its possible establishment in European waters. ICES Journal of Marine Science.

[63] Arnold M, Teagle H, Brown MP, Smale DA (2016). The structure and diversity of epibiotic assemblages associated with the invasive kelp *Undaria pinnatifida* in comparison to native habitat-forming macroalgae on a subtidal temperate reef. Biological Invasions.

[64] Burrows MT (2012). Influences of wave fetch, tidal flow and ocean colour on subtidal rocky communities. Marine Ecology Progress Series.

[65] Connell SD (2001). Urban structures as marine habitats: an experimental comparison of the composition and abundance of subtidal epibiota among pilings, pontoons and rocky reef. Marine Environmental Research.

[66] Marzinelli EM (2012). Artificial structures influence fouling on habitat-forming kelps. Biofouling.

[67] Sogn Andersen G, Steen H, Christie H, Fredriksen S, Moy FE (2011). Seasonal Patterns of Sporophyte Growth, Fertility, Fouling, and Mortality of Saccharina latissima in Skagerrak, Norway: Implications for Forest Recovery. Journal of Marine Biology.

[68] Guzinski Jaromir, Ballenghien Marion, Daguin-Thiébaut Claire, Lévêque Laurent, Viard Frédérique (2018). Population genomics of the introduced and cultivated Pacific kelp Undaria pinnatifida : Marinas-not farms-drive regional connectivity and establishment in natural rocky reefs. Evolutionary Applications.

[69] Epstein G, Smale DA (2018). Between-habitat variability in the population dynamics of a global marine invader may drive management uncertainty. Marine Pollution Bulletin.

[70] Chase JM (2014). Spatial scale resolves the niche versus neutral theory debate. Journal of Vegetation Science.

[71] Carboneras C (2018). A prioritised list of invasive alien species to assist the effective implementation of EU legislation. Journal of Applied Ecology.

[72] Lowe, S., Browne, M., Boudjekas, S. & De Poorter, M. *100 of the World’s Worst Invasive Alien Species*. 11 pp (The Invasive Species Specialist Group (ISSG) a specialist group of the Species Survival Commission (SSC) of the World Conservation Union (IUCN), 2000).

[73] Bates D, Machler M, Bolker B, Walker S (2015). Fitting Linear Mixed-Effects Models Using lme4. Journal of Statistical Software.

[74] Revelle, W. *psych: Procedures for Psychological*, *Psychometric*, *and Personality**Research*. (Northwestern University, 2017).

[75] R Core Team. R: A Language and Environment for Statistical Computing. (Vienna, Austria, 2017).

[76] De Rosario-Martinez, H. phia: Post-Hoc Interaction Analysis (2015).

[77] Clarke, K. R., Gorley, R. N., Somerfield, P. J. & Warwick, R. M. *Change in marine communities: an approach to statistical analysis and interpretation*, *3rd Edition* (2014).

[78] Wickham, H. & Francois, R. dplyr: A Grammar of Data Manipulation (2015).

[79] Wickham, H. *gplot2: Elegant Graphics for Data Analysis*. (Springer-Verlag New York, 2009).

