# Supplementary material for: Identifying niche and fitness dissimilarities in invaded marine macroalgal canopies within the context of contemporary coexistence theory
Source: Sci Rep. 2019 Jun 19;9:8816. doi: 10.1038/s41598-019-45388-5 (PMC6584561; doi:10.1038/s41598-019-45388-5)
Supplement: Supplementary file 1 — Supplementary Material [file 41598_2019_45388_MOESM1_ESM.pdf]

SUPPLEMENTARY INFORMATION

Identifying niche and fitness dissimilarities in invaded marine macroalgal canopies within the context of contemporary coexistence theory.

Graham Epstein, Stephen J. Hawkins & Dan A. Smale

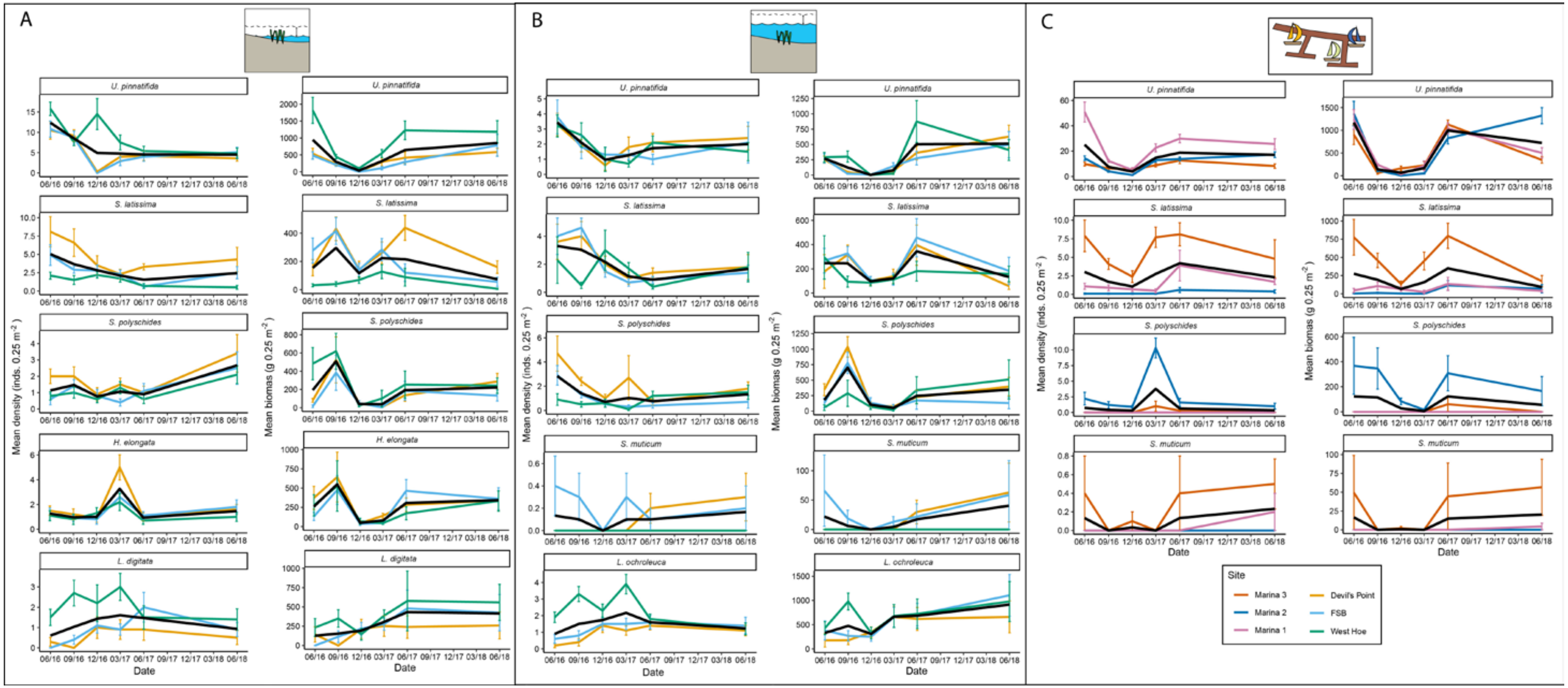

Fig S1 Mean density (L) and biomass (R) of canopy-forming macroalgae per 0.25 m^2 (± standard error) for each site at every sampling event. Data are separated by habitat – intertidal reef (A), subtidal reef (B) and marinas (C). Black lines indicate average across sites.

Table S1 Results from PERMANOVA models testing for difference in composition of canopy-forming macroalgae between years. Each habitat was analysed separately, and models were constructed both on count and biomass data. Site was also included as a random factor in all models. The degrees of freedom (df), pseudo F-value (F) and p-value (*p)* are shown for each coefficient. Significant coefficients shown in bold (α=0.05).

		Intertidal reef		Subtidal reef		Marina	
Coefficient	df	F	p	F	p	F	p
Biomass							
Year	2	2.27	0.052	0.72	0.574	1.17	0.335
Site	2	**3.75**	**0.003**	1.79	0.134	**20.00**	**<0.001**
Density							
Year	2	**5.13**	**<0.001**	2.02	0.111	1.45	0.205
Site	2	**5.15**	**<0.001**	2.29	0.074	**27.03**	**<0.001**

Table S2 Post-hoc pairwise PERMANOVA on the composition of canopy-forming macroalgae (by density) in the intertidal habitat, testing for the effect of year. t value (t) and p-value (*p)* are shown for each pairwise comparison. Significant coefficients shown in bold (α=0.05).

Years	t	p
2016, 2017	**2.26**	**0.006**
2016, 2018	**3.09**	**<0.001**
2017, 2018	1.46	0.136

Table S3 SIMPER analysis on composition of canopy-forming macroalgae, selecting only significant pairwise contrasts indicated from post-hoc pairwise PERMANOVA (Table S2). Average proportion of each species (square root transformed) are shown, with their percentage contribution to the dissimilarity when compared to 2016 (Contrib %).

	2016	2017		2018	
Species	Av. Prop	Av. Prop	Contrib (%)	Av. Prop	Contrib (%)
S. latissima	4.21	2.90	22.64	2.97	21.98
L. digitata	0.96	2.95	22.38	2.22	17.47
U. pinnatifida	7.58	5.97	20.5	5.53	20.67
H. elongata	1.95	2.49	17.96	3.07	19.08
S. polyschides	1.90	2.06	16.51	3.84	20.81

Table S4 Results from ANOVA models on density or percent cover of canopy-forming macroalgae, testing for the effects of substrate (New or Old) and Date (sampling month) on marina pontoons. F-values (F) and p-values (p) shown for each coefficient and species separately. Significant coefficients shown in bold (α=0.01).

		U. pinnatifida		*S. latissima*		*L. digitata*	
Coefficient	df	F	p	F	p	F	p
Density							
Date	14	**103.1**	**<0.001**	**11.2**	**<0.001**	**2.5**	**0.004**
Substrate	1	**221.0**	**<0.001**	**20.1**	**<0.001**	**151.6**	**<0.001**
Date*Substrate	14	**39.7**	**<0.001**	**7.5**	**<0.001**	**2.3**	**0.007**
Canopy-cover							
Date	14	**86.9**	**<0.001**	**6.4**	**<0.001**	**3.1**	**<0.001**
Substrate	1	**217.9**	**<0.001**	0.7	0.408	**142.4**	**<0.001**
Date* Substrate	14	**22.4**	**<0.001**	**4.5**	**<0.001**	**3.0**	**<0.001**

Table S5 Pairwise tests for density or percent cover of canopy-forming macroalgae, testing for differences between new and old marina substrates at each sampling event. F-values (F) shown with Holm adjusted p-values (p) for each pairwise contrast. Significant coefficients shown in bold (α=0.01).

	U. pinnatifida		*S. latissima*		*L. digitata*	
Date	F	p	F	p	F	p
Density						
03/2016	**118.05**	**<0.001**	7.51	0.071	<0.01	1.000
06/2016	**393.13**	**<0.001**	**12.69**	**0.006**	<0.01	1.000
10/2016	**170.57**	**<0.001**	2.29	0.931	**17.58**	**0.001**
12/2016	**79.16**	**<0.001**	0.05	1.000	**17.64**	**0.001**
03/2017	2.84	0.948	0.07	1.000	**19.57**	**<0.001**
04/2017	0.13	1.000	1.31	1.000	**20.60**	**<0.001**
05/2017	0.61	1.000	**19.05**	**<0.001**	**21.02**	**<0.001**
06/2017	0.17	1.000	**35.64**	**<0.001**	**18.94**	**<0.001**
07/2017	1.17	1.000	**20.70**	**<0.001**	**12.42**	**0.004**
08/2017	0.15	1.000	7.19	0.075	**16.68**	**0.001**
09/2017	1.91	1.000	**15.73**	**0.001**	**13.82**	**0.002**
10/2017	1.36	1.000	2.89	0.734	8.22	0.029
11/2017	3.68	0.631	0.25	1.000	5.52	0.082
12/2017	2.67	0.948	0.06	1.000	8.22	0.029
01/2018	1.12	1.000	<0.01	1.000	3.91	0.151
Canopy-cover						
03/2016	10.08	0.020	8.79	0.048	<0.01	1.000
06/2016	**313.68**	**<0.001**	8.53	0.050	<0.01	1.000
10/2016	**123.89**	**<0.001**	6.95	0.105	7.85	0.037
12/2016	**28.94**	**<0.001**	1.89	1.000	**13.06**	**0.004**
03/2017	5.60	0.157	0.52	1.000	**25.82**	**<0.001**
04/2017	**16.23**	**0.001**	<0.01	1.000	**31.16**	**<0.001**
05/2017	3.27	0.439	0.97	1.000	**18.77**	**<0.001**
**06/2017**	**8.09**	**0.047**	10.67	0.020	**21.06**	**<0.001**
**07/2017**	**10.22**	**0.020**	5.03	0.241	**13.47**	**0.003**
08/2017	2.49	0.468	6.74	0.106	**19.82**	**<0.001**
09/2017	3.23	0.439	**13.26**	**0.006**	**15.17**	**0.002**
10/2017	4.46	0.257	0.34	1.000	8.09	0.037
11/2017	1.00	0.955	0.13	1.000	3.02	0.338
12/2017	0.88	0.955	0.01	1.000	3.87	0.257
01/2018	<0.01	0.984	0.06	1.000	2.69	0.338

Table S6 Results from ANOVA models on density or percent cover of canopy-forming macroalgae, testing for the effects of substrate (New or Old) and Date (sampling month) on intertidal reef. F-values (F) and p-values (p) shown for each coefficient and species separately. Significant coefficients shown in bold (α=0.01).

		U. pinnatifida		*S. latissima*		*L. digitata*		*S. polyschides*		*H. elongata*	
Coefficient	df	F	p	F	p	F	p	F	p	F	p
Density											
Date	10	**39.2**	**<0.001**	2.3	0.022	2.4	0.018	**13.3**	**<0.001**		
Substrate	1	**65.6**	**<0.001**	4.4	0.039	**453.7**	**<0.001**	**24.5**	**<0.001**		
Date *Substrate	10	**22.2**	**<0.001**	1.0	0.441	**7.6**	**<0.001**	**13.6**	**<0.001**		
Canopy-cover											
Date	10	**19.9**	**<0.001**	1.8	0.069	**5.4**	**<0.001**	**11.0**	**<0.001**	**17.4**	**<0.001**
Substrate	1	**28.3**	**<0.001**	2.3	0.137	**309.1**	**<0.001**	**29.0**	**<0.001**	**109.9**	**<0.001**
Date* Substrate	10	**6.2**	**<0.001**	0.6	0.788	**4.0**	**<0.001**	**11.0**	**<0.001**	**4.4**	**<0.001**

Table S7 Pairwise tests for density or percent cover of canopy-forming macroalgae, testing for differences between new and old intertidal substrates at each sampling event. F-values (F) shown with Holm adjusted p-values (p) for each pairwise contrast. Significant coefficients shown in bold (α=0.01).

	U. pinnatifida		*S. latissima*		*L. digitata*		*S. polyschides*		*H. elongata*	
Date	F	p	F	p	F	p	F	p	F	p
Density										
03/2017	0.99	1.00	3.15	0.805	**46.96**	**<0.001**	<0.01	1.000		
05/2017	191.34	**<0.001**	0.85	1.000	**40.65**	**<0.001**	**147.93**	**<0.001**		
06/2017	46.19	**<0.001**	0.90	1.000	**143.82**	**<0.001**	**11.79**	**0.010**		
08/2017	49.07	**<0.001**	2.61	0.998	**84.53**	**<0.001**	0.67	1.000		
10/2017	0.25	1.000	5.59	0.231	**62.89**	**<0.001**	<0.01	1.000		
12/2017	0.00	1.000	0.18	1.000	**63.37**	**<0.001**	<0.01	1.000		
02/2018	0.15	1.000	0.23	1.000	**27.89**	**<0.001**	<0.01	1.000		
03/2018	<0.01	1.000	0.23	1.000	**20.00**	**<0.001**	<0.01	1.000		
04/2018	0.01	1.000	0.23	1.000	**22.29**	**<0.001**	<0.01	1.000		
06/2018	<0.01	1.000	0.47	1.000	**11.88**	**0.002**	<0.01	1.000		
07/2018	<0.01	1.000	0.12	1.000	5.86	0.018	<0.01	1.000		
Canopy-cover										
03/2017	0.51	1.000	2.00	1.000	**67.03**	**<0.001**	0.00	1.000	**16.94**	**0.001**
05/2017	**39.54**	**<0.001**	0.01	1.000	**52.64**	**<0.001**	**147.93**	**<0.001**	**36.35**	**<0.001**
06/2017	**33.26**	**<0.001**	0.58	1.000	**44.73**	**<0.001**	**11.79**	**0.010**	**54.85**	**<0.001**
08/2017	**15.71**	**0.002**	1.23	1.000	**39.05**	**<0.001**	0.67	1.000	**16.86**	**0.001**
10/2017	0.44	1.000	3.19	0.868	**42.04**	**<0.001**	<0.01	1.000	9.86	0.018
12/2017	0.27	1.000	0.30	1.000	**19.27**	**<0.001**	<0.01	1.000	0.94	0.576
02/2018	0.24	1.000	0.23	1.000	**18.45**	**<0.001**	<0.01	1.000	2.19	0.575
03/2018	<0.01	1.000	0.25	1.000	**35.42**	**<0.001**	<0.01	1.000	1.71	0.576
04/2018	0.44	1.000	0.05	1.000	**21.49**	**<0.001**	<0.01	1.000	1.74	0.576
06/2018	<0.01	1.000	0.11	1.000	4.91	0.0603	<0.01	1.000	7.94	0.038
07/2018	<0.01	1.000	0.55	1.000	3.83	0.0603	<0.01	1.000	4.30	0.211
